# The Multifunctional Faces of T-Cell Intracellular Antigen 1 in Health and Disease

**DOI:** 10.3390/ijms23031400

**Published:** 2022-01-26

**Authors:** Andrea Fernández-Gómez, José M. Izquierdo

**Affiliations:** Centro de Biología Molecular Severo Ochoa, Consejo Superior de Investigaciones Científicas, Universidad Autónoma de Madrid (CSIC/UAM), 28049 Madrid, Spain; afernandez@cbm.csic.es

**Keywords:** TIA1, gene-expression control, cellular processes, pathophysiological conditions

## Abstract

T-cell intracellular antigen 1 (TIA1) is an RNA-binding protein that is expressed in many tissues and in the vast majority of species, although it was first discovered as a component of human cytotoxic T lymphocytes. TIA1 has a dual localization in the nucleus and cytoplasm, where it plays an important role as a regulator of gene-expression flux. As a multifunctional master modulator, TIA1 controls biological processes relevant to the physiological functioning of the organism and the development and/or progression of several human pathologies. This review summarizes our current knowledge of the molecular aspects and cellular processes involving TIA1, with relevance for human pathophysiology.

## 1. TIA1: Gene, Isoforms and Protein Structure

**Figure 1:**
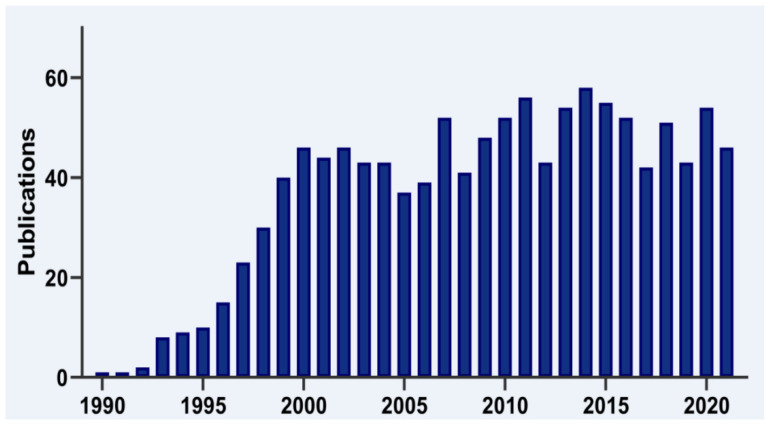
Timeline of TIA1 publications. Histogram representation of the timeline of literature, searching for TIA1/TIA-1 terms in the PubMed/MEDLINE database.

T-cell intracellular antigen 1 (TIA1) is a member of the RNA-binding protein (RBP) family. It is now 30 years since the first report on the cloning and characterization of TIA1 was published by the laboratory of Paul Anderson and colleagues (Figure 1), who identified it as a component of cytotoxic T lymphocyte granules involved in DNA fragmentation [1]. Since its discovery, many research groups have provided insights into the role of this multifunctional master regulator in human molecular and cellular biology, physiology and pathology, which is reflected by a steadily increasing number of publications in the PubMed database (Figure 1).

**Figure 2:**
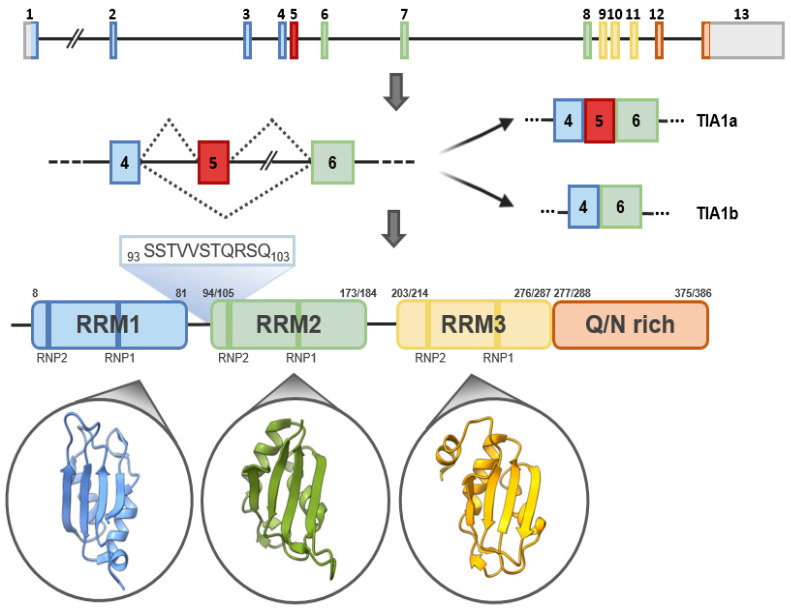
TIA1: gene, isoforms and protein structural details. Schematic representation of human TIA1 gene. Organization of exons and introns of the main isoforms, a and b, generated by alternative splicing, as well as of functional domains of TIA1. TIA1 contains three RNA-recognition motifs (RRMs) and an auxiliary domain rich in asparagine and glutamine (Q/N-rich domain). The amino-acid sequences that differentiate TIA1 a and b isoforms are shown in green. The secondary-tertiary structures of each of the three RRMs are highlighted in the corresponding circles.

The human TIA1 gene is located on chromosomal region 2p13 and consists of 13 exons that, by alternative splicing, can give rise to two major isoforms. At the structural level, TIA1 is similar to other classical RBPs, consisting of three RNA-recognition motifs (RRMs) and a low-complexity glutamine and asparagine (Q/N)-rich carboxyl-terminal domain, which favors protein-protein interactions [2] (Figure 2). In addition, two ribonucleoprotein-forming consensus peptide sequences are conserved within each RRM: RNP1 (8 amino acids), and RNP2 (6 amino acids) [1,2] (Figure 2). Of its 13 exons, exons 1–4 encode RRM1, 5–8 RRM2 and 9–11 RRM3, and exons 12 and 13 encode the C-terminal domain and the 3′-untranslated region (3′-UTR) of its mature messenger RNA (mRNA). Exon 5, which encodes 11 amino acids at the beginning of RRM2, is subject to alternative splicing, which gives rise to TIA1a (43 kDa, including the exon) and TIA1b (40 kDa, omitting the exon) isoforms [1,2] (Figure 2). In the absence of an RNA to bind to, the three RRMs of TIA1 behave as independent structural modules connected by peptide bridges, with almost no intramolecular interaction. Binding to RNA triggers the compaction of the protein domains to form a more rigid RNA-protein complex [3,4].

The Anderson laboratory later identified TIA1-related/like protein (TIAR/TIAL1), which shares 85% amino-acid sequence similarity with TIA1 in the amino-terminal region but only 51% in the C-terminal region, the expression of which also promotes DNA fragmentation [5,6].

## 2. Evolutionary Conservation and Cell/Tissue-Dependent Expression

**Figure 3:**
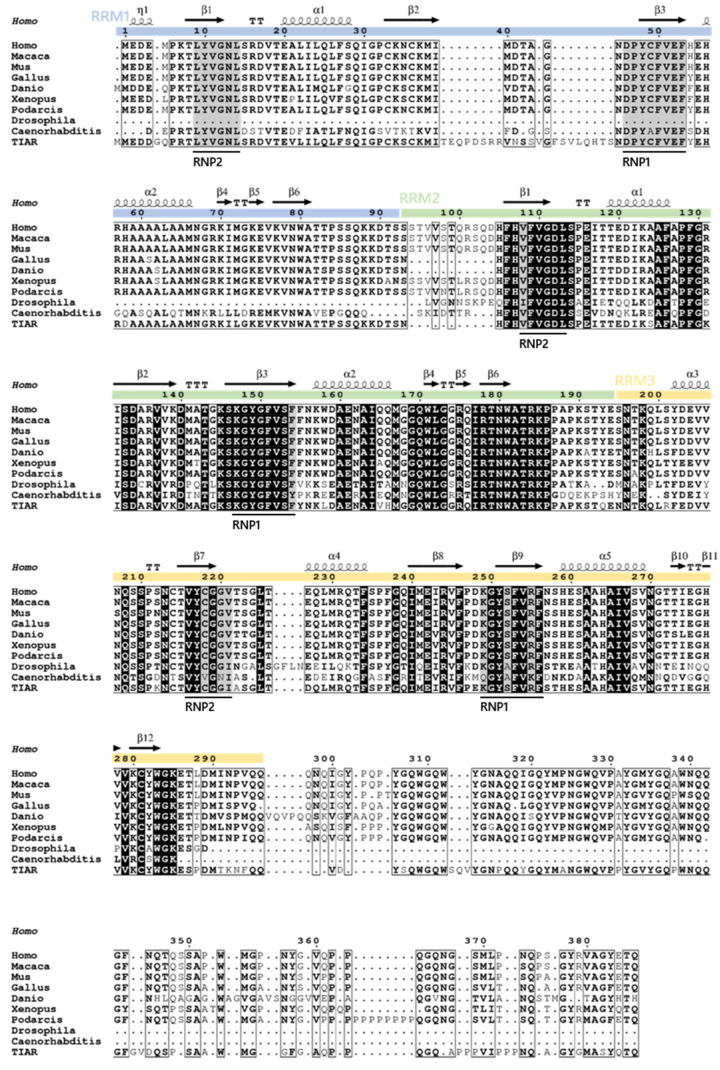
Profound conservation of TIA1 orthologs. Sequence alignment of TIA1 from different species, as well as human TIAR. Original sequences used in the analysis: *Homo sapiens* (Human) TIA1, NP_071505.2; *Macaca mulatta* (Rhesus monkey) TIA1, NP_001248687.1; *Mus musculus* (Mouse) TIA1, NP_035715.1; *Gallus gallus* (Rooster) TIA1, NP_001244132.1; *Danio rerio* (Zebrafish) TIA1, NP_997793.1; *Xenopus tropicalis* (Western clawed frog) TIA1, NP_989276.1; *Podarcis muralis* (Lizard) TIA1, XP_028597015.1; *Drosophila melanogaster* (Fruit fly) DmeI, NP_001303550.1; *Caenorhabditis elegans* (Nematode) RRM domain-containing protein, NP_495121.1; *Homo sapiens* (Human) TIAR, NP_001029097.1. Positions with strictly conserved residues show a black background with white letters and the ones with well conserved residues have a grey background, with the conserved residues in bold letters. Residues conserved between groups are boxed. The secondary structure of the protein, as well as the RRM domains, are shown above the sequence.

Alignment of the primary protein sequence of TIA1 with TIAR and orthologs from different vertebrate species, *Caenorhabditis elegans* and *Drosophila melanogaster*, illustrates the high degree of shared identity (Figure 3). This, together with the existence of structural and functional orthologs both in vertebrates and in invertebrate species, underlines the biological importance of these proteins, which are conserved from the earliest ancestors. However, it is the RRMs that show the highest degree of similarity, with RRM2 (the most involved in RNA-protein interactions) being the most conserved among orthologs, implying strong functional conservation of the mechanism of action of these proteins. By contrast, the C-terminal prion-like domain is much less conserved, likely as a result of a lower selection pressure. Nevertheless, there is a propensity for Q-rich motifs to exist at this end, with few exceptions, including *D. melanogaster* and *C. elegans* [7]. Included among the structural and/or functional orthologs of TIA1 proteins in non-vertebrate taxonomic groups are Rox8, present in *D. melanogaster* [8]; UBP1 and RBP45/47, present in plants [9,10]; Nam8p [11,12,13] and PUB1 [14,15], present in yeast; Ngr1/Rbp1 [16], present in fungi; and SfTRN-1 and BmTRN-1, present in worms [17,18].

What is the need for two structurally and functionally similar proteins (TIA1 and TIAR)? Wang and colleagues argue that this redundancy leads to increased robustness in the regulation of gene expression, although the two proteins may differ in terms of their interactions with other proteins or RNAs and thus regulate some biological processes differently. They may even be post-translationally modified differently, allowing distinct cell-signaling pathways to regulate the activity of TIA proteins and their isoforms. Moreover, the expression pattern of TIA proteins could be distinctly modulated in different tissues [19].

**Figure 4:**
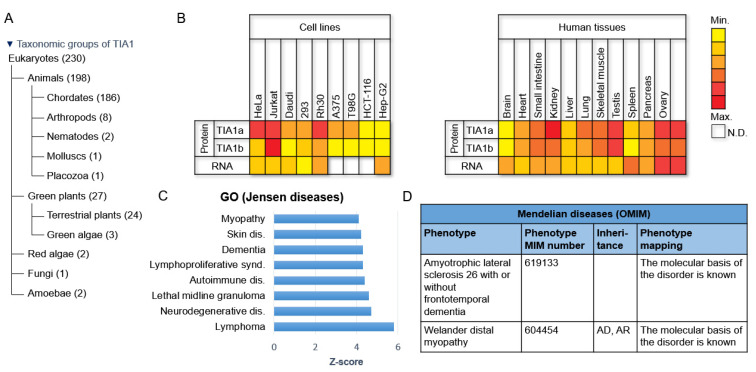
Phylogenetic trees, cellular- and tissular-expression profiling, and Jensen and Mendelian diseases associated with TIA1. (**A**) Taxonomic groups of TIA1 and TIAR orthologs according to the NCBI (National Center for Biotechnology Information) database. (**B**) Expression patterns of TIA1 in human cell lines and in human and mouse tissues. HeLa (uterine carcinoma), Jurkat (T lymphoma), Daudi (B lymphoma), HEK293 (human embryonic kidney), Rh30 (bone marrow rhabdomyosarcoma), A375 (melanoma), T98G (glioblastoma), HCT-116 (colon carcinoma), Hep-G2 (liver carcinoma). (**C**) Gene Ontology (GO) of human diseases related to TIA expression, according to Jensen’s classification. (**D**) Catalog of genetic disorders associated with TIA1 expression based on the Online Mendelian Inheritance in Man (OMIM) database.

TIA1 is virtually ubiquitous. Although discovered in the immune system, it is expressed in many other cells and tissues (Figure 3 and Figure 4A) [1,2,3,20], with cell- and tissue-specific expression patterns for both RNA and protein isoforms, implying the existence of different levels of regulation [20,21]. In humans, TIA1 is expressed in the brain, liver and spleen; however, its expression is significantly greater in the kidney and gonads. The TIA1a isoform is favored in the kidney, ovaries, lungs and spleen [20]. Mice exhibit a somewhat different expression pattern of TIA1 compared to humans (Figure 4B). Although mRNA is evident in almost all tissues, the protein isoforms are predominantly expressed in the brain, spleen and testes and are barely detected in the heart, skeletal muscles or kidney [6] (Figure 4B). The lack of correlation between mRNA and protein levels suggests the existence of post-transcriptional regulatory mechanisms that modulate TIA1 expression. In addition to being linked to tissue type, TIA1 expression is also age-dependent, with a loss of expression with aging [22], indicating the biological relevance of this protein from early embryonic development to aging (Figure 4C,D). To date, massive omics approaches have expanded the known expression patterns associated with TIA1 in many human cells and tissues [23].

Under steady-state conditions, TIA1 is mainly localized in the nucleus (showing nucleolar exclusion) and, to a lesser extent, in the cytoplasm. TIA1 translocates from the nucleus to the cytoplasm under stress conditions [24]. Although it does not possess a specific motif for nuclear localization, the RRM2 domain and the first 50 amino acids of the Q/N-rich domain appear to be responsible for its accumulation in the nucleus, mediated by a Ran-GTP and CRM1-dependent process, the major nuclear import pathway [25]. Export to the cytoplasm appears to be mediated by the RRM3 domain and its binding to RNAs at adenosine and uridine-rich sites (ARE: AU-rich element sequences) located in the 3′-UTR regions of mRNAs. It should be noted that when transcription is inhibited, for whatever reason, TIA1 accumulates in the cell cytoplasm [25] (Figure 2).

## 3. Regulation of Gene Expression

**Figure 5:**
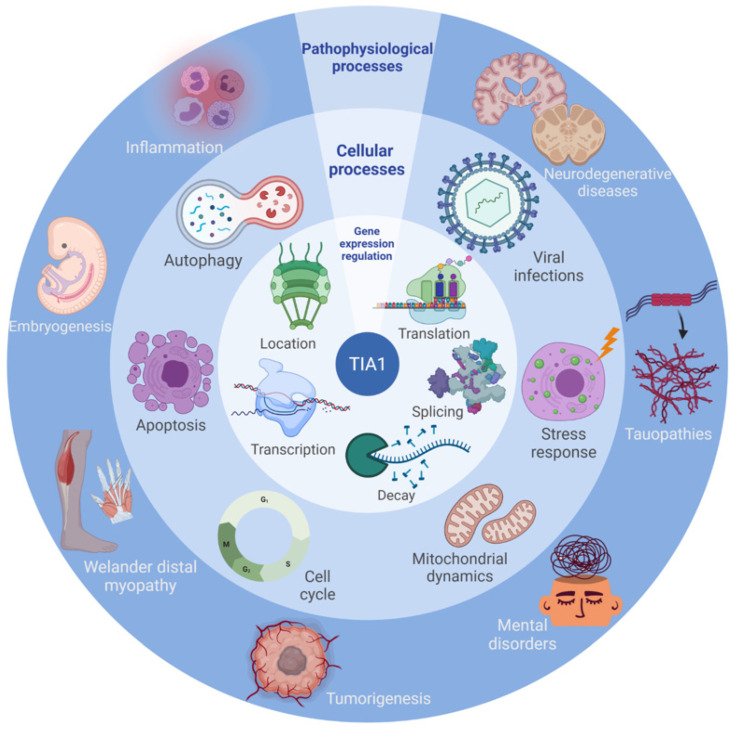
The hallmarks of TIA1 on gene-expression regulation/flux, as well as cellular and pathophysiological processes. Schematic representation of the main processes of gene-expression regulation, cellular events and pathophysiological processes associated with TIA1 expression. This figure was created with BioRender.com (accessed on 20 December 2021).

Gene expression in eukaryotic cells is an orderly process comprising: DNA replication, transcription; post-transcriptional processes, such as processing/splicing of pre-mRNAs; and transport, stability and translation of mature mRNAs. Additionally, post-translational regulatory mechanisms modulate the function, stability and fate of proteins. By virtue of its properties of binding to cellular RNA/DNA, TIA1 participates in the regulation of many of these processes. In the presence of RNA, it binds to small fragments of 3–11 uracil-rich ribonucleotides [21], but it is also known to have capacity to bind to equivalent DNA sequences through the RRM2 [26] (Figure 5).

### 3.1. Transcriptional Rates

**Figure 6:**
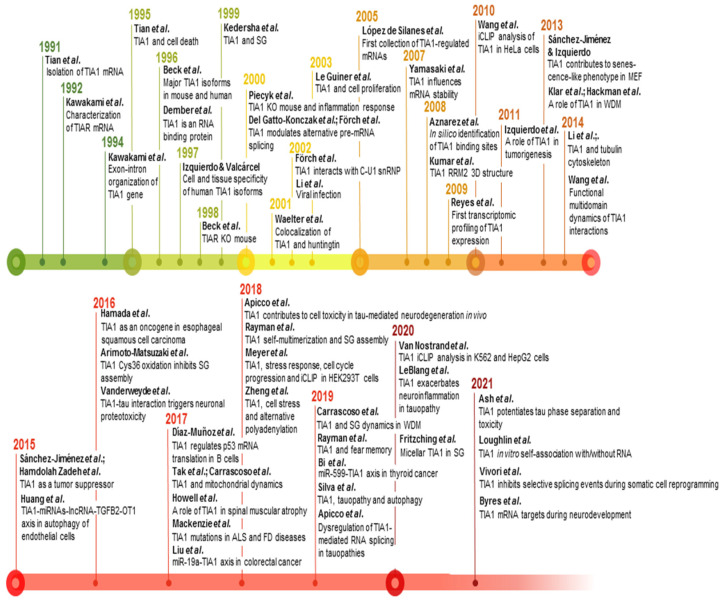
Timeline and milestones of TIA1 research. Milestones in the study of TIA1/TIA-1 found in PubMed/MEDLINE database. The references to build this figure are the following: [1,2,3,4,5,6,13,19,20,21,24,31,33,34,35,36,37,38,40,42,44,46,73,77,83,84,85,86,87,88,89,90,91,92,93,94,95,96,97,98,99,100,101,102,103,104,105,106,107,108,109,110,111,112,113,114].

TIA1 was first implicated in transcriptional regulation in 2007, when it was described that the C-terminal domain of RNA polymerase II interacted with components of the spliceosome processing factor snRNP U1 and other proteins, including TIA1 [27] (Figure 5 and Figure 6). These interactions facilitated simultaneous transcription and processing/splicing of cellular pre-mRNAs. Once TIA1 proteins are bound to DNA, RNA sequester them by affinity, facilitating dynamic post-transcriptional regulation [27]. The mechanism of action is speculated to involve the slowing of RNA polymerase II [26], as well as the end of transcription and associated 3′ end processing [28]. Genes such as *COL2A1* (procollagen, type II) [24], *IGFBP*-3 (insulin-like growth-factor binding protein-3) [29] or testicular *PACAP* (pituitary adenylate cyclase-activating polypeptide) [30] seem to be preferentially regulated by this pathway. A comprehensive analysis of the transcriptome of HeLa cells with transient reduction in TIA protein expression revealed changes in a large number of mRNAs associated with cellular processes such as inflammation, cell signaling, suppression of immune response, angiogenesis, apoptosis, metabolism and cell proliferation [31]. In this context, the participation of these proteins in the direct modulation of *PTGS2* (prostaglandin-endoperoxide synthase 2) and IL-6 (interleukin 6) gene transcription was demonstrated [31]. Similar results were obtained in the study of the transcriptome of mouse neuronal tissues (spinal cord and cerebellum) in the absence of TIA1 expression [32].

### 3.2. Post-Transcriptional Control

One of the nuclear functions of TIA1 is to regulate the alternative splicing (and also constitutive splicing) of some pre-mRNAs to favor the inclusion [19,33,34,35] or selective exclusion [36] of exons (Figure 5 and Figure 6). For splicing to occur, the spliceosome must first be assembled, which begins with the recognition of the 5′ intronic splicing site (5′ss) by the nuclear ribonucleoprotein U1 (snRNP U1). It is in this context that TIA1 exerts its function. To promote exon inclusion, TIA1 binds to uridine-rich RNA sequences located near both constitutive and alternative 5′ss with weakly conserved consensus sequences for U1 snRNA hybridization and U1 snRNP anchoring and subsequently binds to the U1-C protein of the U1 snRNP [13,19,33,34,35,36,37,38,39]. In this process, RRM2 binds to the uridine-rich regions near the 5′ss of the intron, favored by RRM3, and the Q/N-rich domain binds to the N-terminal region of the U1-C subunit of the U1 snRNP by an RRM1-favored process [38,39]. Its role as a splicing repressor is linked to competition for uridine-rich sequences within the polypyrimidine-rich regions located at positions near the 3′ss of introns to interfere with auxiliary-factor (perhaps U2AF65) binding by preventing U2 snRNP recruitment [35,36].

The cellular relevance of TIA1 in the regulation of alternative and constitutive splicing was confirmed through the establishment of a binding map of these regulators to RNA in HeLa cells using in vivo irradiation of cells with ultraviolet light and subsequent immunoprecipitation of RNA-protein complexes (termed iCLIP) [19]. Results from this seminal study have been corroborated by recent data in other cell lines using PARCLIP and eCLIP approaches [34,35]. These approaches demonstrate that TIA1 preferentially binds to ARE sequences located in introns, in 5′ and especially 3′ UTR positions––preferably UUUUA or AUUUU tandem pentamers––of mRNAs, as well as to non-coding RNAs. From these studies, it is estimated that TIA1 modulates gene expression of 2–5% of the human genome.

### 3.3. Regulation of Translation

TIA1 can modulate cellular translation by limiting the availability of the ribosomal machinery and the translational efficiency of specific cellular mRNAs, either under conditions of stress, to safeguard cell viability, or under conditions of homeostasis [19,40,41,42,43,44] (Figure 5 and Figure 6).

In the absence of stress, 48S translation initiation complexes, which include the ternary translation-initiator complex eIF2-GTP-tRNAMet, the small subunit of the ribosome, other translation initiation factors (eIFs) and mRNA, are formed in the cell. When translation is initiated, eIF5 causes hydrolysis of eIF2-associated GTP. The 60S subunit of the ribosome then binds, displacing some eIFs, and the 80S ribosome is formed. However, under stress, the α-subunit of eIF2 is phosphorylated by a family of kinases such that eIF2B cannot exchange GDP for GTP, eIF2-GTP-tRNAMet levels decrease and translation pre-initiator complexes cannot form properly. At this point, TIA1 associates with the complex formed by eIF4E, eIF4G, eIF4A, eIF4B, eIF3 and the small subunit of ribosome in an anomalous 48S complex. These inactive translation pre-initiator complexes associated with mRNAs accumulate in the cytoplasm and, through the aggregation properties of the Q/N-rich domain of TIA1 and PABP (Poly(A) Binding Protein) proteins, bind together, forming large foci of mRNAs and proteins termed stress granules (SGs) [45,46,47,48]. The formation of SGs favors cell survival in stressful situations, such as during viral infection, fasting or limited amino-acid availability, and oxidative or osmotic stress. In these adverse conditions, the cell enters a state of biological quiescence, inhibiting general translation, which allows energy to be conserved to repair the damage produced by the stressful insult. For example, cells respond to changes in nutrient conditions by regulating the expression of the so-called 5′TOP (5′-Terminal OligoPyrimidine tract) mRNAs, which encode factors for protein biosynthesis, including many ribosomal protein-coding mRNAs. These mRNAs contain an oligopyrimidine (UC)-rich sequence at the 5′-UTR end. It has been suggested that TIA1, through GCN2 activation, binds to these sequences and facilitates mRNA arrest in the SGs under fasting conditions. Thus, reprogramming of the translational machinery and protein expression ensues, favoring cell survival [49,50], although these claims are controversial [51,52]. On the other hand, there are other biological processes for which the formation of SGs is relevant from a physiological perspective––for example, in the differentiation of naïve CD4+ T lymphocytes to cytokine-secreting effector cells. After priming of naïve lymphocytes, the requirements for differentiation and rapid clonal expansion are so high that they trigger nutrient deficiencies and induce stress. Under these conditions, eIF2α is phosphorylated, and cytokine mRNAs accumulate in SGs. Stimulation of lymphocytes leads to dephosphorylation of eIF2α, the disappearance of SGs and the reactivation of cytokine synthesis [53].

SGs are dynamic structures, and most of their components are in a constant flux of assembly and disassembly [54,55,56]. SGs are formed approximately 15 min after the onset of the stressful stimulus, and their formation is reversible, disappearing within a few hours (between 2 and 6 h) of the cessation of the stimulus, provided that it is not lethal [45,46,54]. Microscopic observation of SGs shows that their size is variable and cell-type-dependent, ranging from 150 to 200 nm in stressed rat hippocampal cells [45]; 0.1–0.2 µm in COS cells [57]; or 1–2 µm in HeLa or HEK293 cells [56]. There is some controversy regarding the involvement of TIA1 and/or TIAR in SG formation. It has been suggested that both TIAR and TIA1 are needed [57], yet other authors have shown that the presence of one of the two proteins is sufficient for SG generation [42]. In fact, the results of high-resolution fluorescence microscopy, together with fluorescence recovery after photobleaching experiments, suggest that SGs have two different layers with different components, dynamics and functions: a stable inner structure or core surrounded by a less dense layer or shell. Accordingly, the components of the structure that make up the core would be less dynamic, whereas the components of the shell would be more dynamic. This hypothesis allows us to propose two models to explain the SG assembly process. The first model suggests that SG formation begins with the assembly of the riboproteomic core, followed by rapid growth or assembly of the envelope. The first small SGs would fuse to generate larger SGs through liquid-liquid phase-separation (LLPS) processes. Alternatively, the second model proposes that it is the LLPS states promoted by the formation of translationally repressed riboproteomic complexes that form first, subsequently promoting the separation of droplets that constitute cores in the context of elevated protein concentrations [58,59]. TIA1 would be one of the constituents of the nuclei of SGs [60].

There are many proteins (i.e., RBPs, non-RNA-binding proteins and translation-initiation factors) that have been identified as components of SGs, in addition to RNAs (coding and non-coding). These include the HuR protein (Hu antigen R/Embryonic lethal, abnormal vision, Drosophila-like 1) [60] and some proteins involved in RNA stability/degradation, such as TDP-43 (TAR DNA-binding protein) [61,62], hSMG-1 (phosphatidylinositol 3-kinase-related kinase) [63] and TTP (TrisTetraProlin) [60]. The presence of these proteins alongside TIA1 implies a model of equilibrium between antagonistic factors capable of promoting the degradation or stability of mRNAs located in SGs. Finally, the role of G3BP1 and 2 (GTPase-activating protein 1 and 2) in the formation of some SGs is noteworthy. This protein does not interact with TIA1 per se but contains a self-aggregation domain and is functionally similar to TIA1. An immunoprecipitation study of RNA-protein complexes identified several hundred TIA1-associated mRNAs under heat-shock stress conditions. The study revealed the binding of TIA1 to uridine (U)-rich motifs at 5′ ends and to adenosine-uridine (AU) at 3′ ends of the 3′-UTR region of mRNAs, such as ACTB (actin, beta), CALM2 (calmodulin 2) or CASP7 (caspase 7), among others [42]. Similarly, analysis of TIAR-associated mRNAs under UV stress conditions, without apparent phosphorylation of eIF2α, demonstrated the repression of translation of some translation initiation factors, such as EIF4A, eIF4E and eIF1B, as well as the transcription factor C-MYC, which would favor the inhibition of translation once the stress is relieved, causing the inhibition of global translation of the cell [64]. The involvement of TIA1 in the regulation of mitochondrial cytochrome *c* mRNA translation [42,43] of the transcription factor HIF-1α (hypoxia inducible factor 1, alpha subunit) [65] and of the molecular chaperone Hsp70 (heat-shock protein 70) [66] has also been demonstrated. These results have been confirmed by iCLIP/PARCLIP/eCLIP analysis of TIA1 in other cell models [19,34,35]. It appears, however, that selective translational activation of some mRNAs excluded from SGs occurs under stress conditions [45,56,67].

TIA1 has the ability to globally and/or specifically inhibit the translation of cellular mRNAs in stress-independent conditions by binding to specific sequences located in the 5′- and/or 3′-UTR region of mRNAs [19]. The first evidence of this phenomenon was documented in activated macrophages in which deficiency of TIA1 led to an increase in the levels of the pro-inflammatory protein TNFα (tumor necrosis factor α) without affecting the relative levels of its mRNA [40,68,69]. This occurred through the binding of TIA1 to AU-rich sequences in the 3′UTR region of TNFα mRNA [68,70], a process that has also been described for other cellular mRNAs [19,35,41].

### 3.4. Stability of mRNAs

mRNA turnover is the process by which an mRNA is degraded before or after translation. This can occur at the 5′ end of the mRNA through decapping by the activity of DCP1 and DCP2 (dipeptidyl carboxypeptidase 1 and 2) and the exonuclease XRN1 (5′-3′ exoribonuclease 1) [71] or at the 3′ end, mediated by the exosome, resulting in deadenylation of the poly(A) tail and recruitment of exonucleases [72]. The mRNA regions involved are the ARE sequences located in the 3′-UTR that favor the binding of TIA1, AUF1 (AU-rich element RNA-binding protein 1), KRSP and TTP, which recruit proteins involved in the degradation process. Binding of TIA1 to these regions favors both mRNA deadenylation and stimulation of 5′cap removal, in a cell-specific and stress-independent manner [35]. By contrast, proteins such as HuR stabilize mRNA, likely because of their inability to recruit the exosome to ARE sequences [73] (Figure 5 and Figure 6). mRNA stability can also be regulated through the binding of microRNAs (miRNAs), which can trigger degradation. miRNAs are small fragments of non-coding RNA, of 19–24 nucleotides, that regulate gene expression by pairing with sequences typically located in the 3′ UTR regions of mRNAs. The interaction of miRNA with RNA stimulates the recruitment of the RISC complex (RNA-induced silencing complex) and mRNA cleavage. Several studies suggest that 20–30% of gene expression is controlled by miRNAs [74]. A large-scale expression-platform study demonstrated that transient knockdown of TIA1 and TIAR in HeLa cells resulted in the overexpression of 29 miRNAs [75]. This result was interpreted as a strategy to counteract the differential expression and cellular phenotypes associated with the downregulation of TIA proteins [75]. Moreover, in cellular microvesicles abundantly expressing TIA1 and representing a mechanism of cellular communication in stem cells, 365 miRNAs were identified, the target mRNAs of which are related to organism development, survival and differentiation, as well as regulation of the immune response [76]. This evidence suggests that TIA1 can activate or repress the transcription of miRNAs through a mechanism that is currently unknown and can interact with them to modulate gene expression. Finally, it is worth noting that non-coding RNAs have been identified, both small RNAs and long, non-coding RNAs, that repress TIA1-associated expression through direct interaction with its mRNA, impacting its translation and/or stability (e.g., miR-19a [77], miR-487a [78], mivaRNAI-138 [79]) or the direct sequestration of TIA1 and other RNAs through a molecular sponge mechanism (e.g., FLJ11812) [80], NORAD [81], MALAT1 [82]).

## 4. Cellular Processes

The multifunctional capacity of TIA1 to modulate gene expression points to an important role of TIA1 in the maintenance of cell homeostasis and in the regulation of several cellular processes with pathophysiological implications (Figure 5 and Figure 6).

### 4.1. Apoptosis

The health of all multicellular organisms depends not only on their ability to produce new cells but also to get rid of unwanted cells (Figure 5). The first experimental evidence for the involvement of TIA proteins in cell-death regulation was provided through the observation that incubation of permeabilized target cells of cytotoxic T lymphocytes with TIA1 triggered nuclear DNA fragmentation [1]. Subsequently, it was found that TIA1 regulates the gene expression of different components of cell-death pathways, allowing for the activation of a signaling cascade that drives apoptosis [24,83,84]. There are, however, other routes by which TIA proteins can modulate cell death, such as the sequestration of RSK2 protein (ribosomal protein S6 Kinase, 90 kDa, polypeptide 3) in SGs under stress conditions, favoring cell survival [115], or favoring/repressing the synthesis of other proteins involved in the regulation and/or execution of the cell-death/survival program [31,32,83,84]. Complete inactivation of TIA1 and TIAR in HEK293 cells also leads to mitotic catastrophe and cell death [34] (Figure 5 and Figure 6).

### 4.2. Autophagy

Autophagy is an evolutionarily conserved mechanism to eliminate dysfunctional cells or their damaged components, promoting cell regeneration and clearance (Figure 5). TIA1 has been shown to interact with Annexin A7, a regulator of its phosphorylation, in endothelial cells, which favors the processing of the pro-autophagic factor FLJ11812 and the expression of autophagy-related protein 13 (ATG13) [85]. There are several well-documented links between autophagy responses, SGs and human pathologies, such as tau-associated pathology [86], Parkinson’s disease and other neurodegenerative disorders (e.g., Alzheimer’s disease (AD), Huntington’s disease (HD), amyotrophic lateral sclerosis (ALS), tauopathies and frontotemporal lobar dementia (FTLD)) [86,116]. Interestingly, mTOR-driven pharmacological stimulation of autophagy attenuates the high-glucocorticoid (GC)-driven accumulation of tau and SG-related proteins, as well as cell death, suggesting a critical interface between autophagy and the response of SG-related proteins in the neurodegenerative potential of chronic stress and GC [85]. These studies provide novel insights into the RNA-protein intracellular signaling pathways regulating the precipitating role of environmental stress and GC on tau-driven brain pathology [86] (Figure 5 and Figure 6).

### 4.3. Cell Proliferation and Cell Cycle

TIA1 and TIAR modulate cell proliferation by promoting or inhibiting cell growth (Figure 5). For example, it has been described that the loss of expression of TIA proteins in DT40 chicken lymphoma cells inhibits their growth [87]. This study also showed that cells need at least one allele of one of the two genes to maintain their viability. In mouse embryonic fibroblasts, knockout of Tia1 or Tial1 results in cell-proliferation defects and stimulates cell death [40,87,88], and simultaneous inactivation of both proteins in HEK293 cells leads to mitotic catastrophe and apoptosis [34]. In contrast, in immortalized or tumor cells, transient or stable downregulation of TIA1, TIAR or both enhances cell proliferation and is associated with a tumorigenic phenotype [31,89]. This has been the case observed in several human cell lines: K562 (myeloma) [117], HCT116 (colorectal carcinoma) [118], HEK293 (embryonic kidney cells) [118] and A549 (adenocarcinoma) [65]. Contrastingly, overexpression of TIA1 in tumor lines leads to a cell-quiescence phenotype that ultimately triggers cell death by apoptosis and autophagy [83,90,91,92]. These data are consistent with the importance of the genetic background, as well as tissue- and isoform-dependent expression of TIA1, which determines its dual functionality as an activator or repressor of the cell cycle and cell proliferation, perhaps depending on the target interactomes (RNAs and proteins) that it modulates as a function of cellular and/or environmental conditions (Figure 5 and Figure 6).

### 4.4. Mitochondrial Dynamics

The mitochondrion is a cytoplasmic organelle unique and essential to eukaryotic cells but with a proteobacteria origin. Mitochondria respond to changes in genetic, metabolic and/or environmental conditions by modifying the dynamics of their morphology and architecture to achieve cellular homeostasis or to initiate mitophagy and/or cell death or apoptosis. TIA1 has been associated with mitochondrial respiration and mitochondrial fission/fusion dynamics [43,88,92,93,94,119]. Mitochondrial respiration is linked to TIA1 expression [43,92,94,119]. Indeed, TIA1 directly regulates the translation of cytochrome c mRNA [43]. Deletion or reduction of TIA1 leads to the accumulation of elongated mitochondria [88,94,119]. Conversely, overexpression of TIA1 favors mitochondrial clustering and division/fission [92,93,94]. The function of TIA1 also appears to be associated with translation of mitochondrial fission factor (MFF) through competition with miRNA-27 to bind to the 3′UTR [94], as well as with OPA1 expression through alternative splicing and differential processing of its isoforms [92,119] (Figure 5 and Figure 6).

### 4.5. Embryonic Development

TIA1 expression is not homogeneous in all mouse embryonic tissues and is greater in neuronal tissues, such as brain and retina [120]. Indeed, docosahexaenoic-acid and arachidonic-acid treatment of mouse neonates favors the development of the nervous system, concomitant with an increase in TIA1 expression [121]. The importance of TIA1 and its orthologs has also been observed in other organisms; for example, in *D. melanogaster*, Rox8 protein expression is increased during the egg-formation process [8]. In *Bombyx mori*, the TIA1 ortholog BmTRN-1 increases the expression of EN86 in the midgut during larvae-pupa metamorphosis [122]. EN86 participates in programmed cell death in the silk gland during metamorphosis [17]. During *Xenopus laevis* embryogenesis, TIA1 is predominantly transcribed during nervous-system development, supporting the notion that TIA1-mediated regulation of gene expression plays an important role in amphibian neuronal development [123]. In *C. elegans*, TIA1 expression is associated with apoptosis of some germ cells to maintain gamete quality [124]. Overall, these data indicate that the temporal and spatial expression of TIA1 during early development is key for the correct establishment of embryogenesis [32,36,88,95].

The relevance of TIA proteins during embryogenesis was addressed in vivo by generating mutant mice deficient in TIA1 [40] and TIAR [96]. Homozygous mice lacking TIA1 and TIAR died before embryonic day (E)7. Experiments in several mouse strains revealed that in the absence of TIA1, 46% of mice died between E16.5 and 3 weeks of age, and surviving mice had no apparent major abnormalities other than an arthritis-associated phenotype [40]. A later study found that these mice had an increased sensitivity to house dust, produced by an exacerbated allergic reaction accompanied by pulmonary inflammation, increased IgE and IgG1 levels and increased transcriptional capacity of Th2/Th17 cytokines in lymph-node cells [125]. By contrast, the phenotype of mice lacking TIAR appears to depend on the mouse strain in which the studies are performed. TIAR deficiency resulted in embryonic lethality in 100% of BALB/c mice and in 90% of C57BL/6 embryos [96]. Crossing BALB/c TIAR+/− mice with C57BL/6 TIAR+/− mice produced 60% embryonic mortality. Of the remainder, half of the mice survived to adulthood but were sterile, with abnormalities in spermatogenesis and oogenesis, as well as in the architecture of the gonads themselves. Other phenotypes included obesity, despite being born with lower body mass, and neurological disorders. In addition, the mice developed cervical cancer as adults [96]. Conversely, in a transgenic mouse model of TIAR overexpression, 77% of embryos had abnormalities at E7.5 [126]. The phenotypic differences observed between mice with inactivation of TIA1 and/or TIAR indicate that they may cooperate or act independently during early embryogenesis (Figure 5 and Figure 6).

### 4.6. Inflammation

Inflammation is one of the immune system’s defense mechanisms against the presence of dangerous agents or toxic products (i.e., bacteria, viruses, damaged cells, and/or irritant reagents). The essential function of this complex cellular response is to eliminate and repair cellular damage. The involvement of TIA1 in the molecular mechanisms associated with inflammatory processes was discovered in the early 2000s [40] (Figure 5 and Figure 6). The differential expression of TIA1 provokes inflammatory scenarios in different systems mainly associated with cytotoxicity generated via CD8+ T lymphocytes and other cells [1,127]. This involves transcriptional and post-transcriptional regulation of gene expression of immune-system components and effector molecules (IL1-β, IL-6, IL-8 and TGF-β) [31,40,42,69,70,73] and of the vascular system, including early angiogenic targets, (i.e., PTGS/COX-2, VEGF and ELAVL1/HuR) [31,41,42,90]. In many cases, TIA1-mediated inflammatory responses have pathophysiological consequences associated with disorders such as arthritis, phagocytosis/autophagy, atherosclerosis, angiogenesis, neuroinflammation, obesity, myopathy, tauopathy and cancer [32,40,92,93,97,128,129,130,131] (Figure 5 and Figure 6). The pro-inflammatory cytokine TNF-α is a fundamental factor in the activation of proliferation and cytotoxicity, as well as in apoptosis. TNF-α is transcriptionally and post-transcriptionally regulated by TIA1, among other RBPs [40,68,69]. TNF-α is involved in many inflammatory processes in the body, including food allergies, rheumatic diseases such as arthritis or lupus, incompatibility in transplantation or difficulties in operations, as well as endometriosis due to cytokine regulation of endometrial stem cells, where TIA1 shows a differential and cyclic concentration [97,129,131]. Additionally, a TIA1-associated neuronal inflammatory phenotype has been described in tauopathies in the P301S mouse model mediated by a cytotoxic process of the central nervous system via TNF-α, IL-6 and IL1-β [97].

TIA1-knockout (KO) mice have a profound inflammatory diathesis and are markedly more susceptible to lipopolysaccharide-induced septic shock than wild-type counterparts [40]. Moreover, TIA1-KO mice develop spontaneous arthritis, associated with pathological overexpression of TNF-α [130]. Thus, the effects of TIA1 are cell-type and transcript-specific. TIA1 acts on both hematopoietic and non-hematopoietic cells to dampen pulmonary inflammation, and it may play an important role in the pathogenesis of bronchial asthma [125]. TIA1 can function as a translational repressor to dampen the production of pro-inflammatory cytokines and enzymes. In fact, by coordinately repressing the translation of multiple inflammatory mediators, TIA1 applies a post-transcriptional “brake” that prevents pathological inflammation [130]. Mechanistically, TIA1 binds to the ARE sequences in the 3′ UTR of mRNAs that encode inflammatory mediators, including TNF-α [40]. TIA1 also targets mRNAs that encode pro-inflammatory enzymes, such as cyclooxygenase-2 (COX-2) [41,132]. Transcriptional profiling indicates that TIA1 presides over a large network of immune-system genes with modulatory roles in synaptic plasticity and long-term memory [98].

### 4.7. Stress Granules

Stress granules are non-membranous aggregates that form from mRNAs arrested at translation initiation and thus contain translation initiation factors, in addition to proteins with both RNA-binding and other functions [58,133] (Figure 5). Similar to other non-membranous intracellular compartments, such as processing bodies or Cajal bodies, SGs are formed by LLPS [99,134] (Figure 5). This implies that within the cell, the regions change their material properties, which become liquid-like and can be differentiated from the rest of the cytoplasm without the need to be separated by a membrane [134]. Interestingly, upon some endogenous or exogenous stress situations, the cell can trigger LLPS of specific proteins, which can also include other proteins as part of these condensates [99,100,134]. Proteins with low-complexity, intrinsically disorganized prion-like domains (PLDs)—as is the case of the C-terminal domain of TIA1—are known to have the ability to trigger LLPS. Indeed, it has been shown that TIA1 can induce LLPS not only in vivo, associated with RNA and other proteins to form SGs [57,101,102,103], but also in vitro by self-aggregating in the presence of RNA or single-stranded DNA, for which it has high affinity [100,101,102,103]. Although these C-terminal PLDs of TIA1 are known to interact with one another during SG formation, remaining disorganized, the precise arrangement of the protein within aggregates is unknown, although a model has been proposed whereby TIA1 gives rise to micellar-like structures [99]. While the sequence-intrinsic features that drive LLPS or control the material properties of the resulting aggregates are not yet fully understood, the existence of a “molecular grammar” has recently been demonstrated, according to which interactions between specific amino acids can either promote or impede the process [134]. Amino-acid properties such as polarity, aromaticity, charge and size strongly influence their ability to interact with other protein regions and trigger LLPS, resulting in SG formation [134] (Figure 5 and Figure 6). To date, methodologies to study SGs functions are mostly based on gain- and loss-of-function analysis of SGs components, confocal and electron microscopy, cell fractionation and proximity-labeling biotinylation to isolate and characterize the SGs. This latest methodology, coupled with omics-based approaches, has identified molecular components associated with these transient granules. Currently, more than 400 proteins have been identified in stress granules, but their compositions vary between cell types and/or stressing conditions, which is in agreement with proteomic approaches used to identify many of the proteins located mammalian SGs [135,136]. Among them, about 50% are RBPs, while the remaining proteins are presumably recruited through protein-protein interactions and are involved in important cellular processes (e.g., cell-cycle progression, cellular signaling pathways, apoptosis), as well as in assembly regulation of SGs. Updated information is available in several databases [135,136], and interestingly, several components have been associated to various cellular processes, for example, cancer-related events (carcinogenesis), suggesting an unknown role of SGs in the formation, growth and/or dissemination (metastasis) of many human tumors.

### 4.8. Viral Infections

The cellular-stress response constitutes one of the first lines of defense against viral infection. Its activation can lead to global translational repression in the host cell, as well as SG formation, processes in which TIA1 is directly involved (Figure 5). Upon infection, PKR kinase phosphorylates eIF2α, inhibiting the translation of both cellular and viral mRNAs, which target SGs while translating proteins involved in cellular-damage repair [137]. This is the case of vesicular stomatitis virus (VSV), which induces the formation of SG-like structures containing TIA1, in addition to viral RNA and proteins [138]; or human enterovirus D68 (EV-D68), in which TIA1 present in SGs participates in the inhibition of viral replication by interacting with viral RNA [139]. TIA1 also binds hepatitis B virus (HBV) post-transcriptional regulatory element (PRE), inhibiting its function [140]. However, evolution has allowed many viruses to evolve mechanisms to evade this antiviral system and to even use the phosphorylation of eIF2α and TIA proteins to their own advantage. For example, poliovirus (PV) can inhibit the formation of SGs containing TIA1, which sequester translation initiation factors during infection [141]. In Sindbis virus (SINV) infection, phosphorylation of eIF2α is required to initiate translation, and the formation of SGs, in which capsid proteins are recruited with the aid of TIA1, promotes protein synthesis [142]. This is also the case for some flaviviruses, such as dengue virus (DENV) and West Nile virus (WNV), which are able to use TIA1 and TIAR as transcription factors to favor the synthesis of their genomes [104,143,144]; and Zika virus (ZIKV), for which it has been proposed that TIA proteins may localize to replication sites during infection, modulating translation and thus affecting viral RNA and protein levels [145,146]. Other examples are human papillomavirus (HPV) [147] and minute virus of mice (MVM) [148], which use TIA proteins to splice their own transcripts; or hepatitis C virus (HCV), in which TIA1 plays a role in genome replication and virus assembly, while TIAR participates in its exit from the cell [149]. Interestingly, TIA1 has been identified as one of the proteins of the severe acute respiratory syndrome coronavirus 2 (SARS-CoV-2) interactome [150] (Figure 5 and Figure 6).

## 5. Pathological Situations

### 5.1. Neuropathologies

#### 5.1.1. Amyotrophic Lateral Sclerosis

Amyotrophic lateral sclerosis (ALS) is characterized by the progressive degeneration of motor neurons in the cerebral cortex and spinal cord, resulting in severe muscle weakness. Although its cause remains unknown, several genetic mutations have been found that could be related to the progression of the disease. Many of them affect RBPs involved in RNA metabolism found in SGs, as is the case of TIA1 [151]. This has led to an increased focus on dysregulation of RNA homeostasis mechanisms and improper functioning of RBPs as causes of ALS pathology. Mackenzie and colleagues identified pathological mutations in the C-terminal, prion-like domain of TIA1 in patients with ALS with or without associated frontotemporal dementia [105], and subsequent studies have expanded the spectrum of known mutations [152,153]. These prion-like domains confer biophysical properties to proteins that allow them to trigger LLPS and produce non-membranous organelles [154,155,156]. Therefore, mutations in the C-terminal domain of TIA1 increase its propensity to produce LLPS and thereby alter the functionality of SGs, slowing down their disassembly. Furthermore, Mackenzie and colleagues found that SGs sequestered TDP-43, although they were not able to demonstrate a colocalization of TIA1 with TDP-43 in the inclusions and failed to find any abnormal accumulation of other RBPs. Nevertheless, they observed the appearance of round, hyaline, Lewy body-like cytoplasmic inclusions in lower motor neurons as a possible hallmark of ALS pathology produced by TIA1 mutations [105,157]. However, subsequent studies showed that the work of Mackenzie and colleagues did not provide sufficient evidence to support a causal role of TIA1 mutations in ALS [158,159]. Recently, it has been observed that late stages of ALS-like disease in mice with Cu/Zn superoxide dismutase (Sod1) mutations show increased TIA1 expression and TIA1-positive SGs in lumbar spinal cord cells, accompanied by an increase in TIA1-Sod1 interactions and a delay in the formation of Sod1-related stress-induced SGs [160]. These results suggest that the interaction of mutant Sod1 with TIA1 could perturb the dynamics of SGs and be a possible pathological mechanism of familial ALS development [160] (Figure 5 and Figure 6).

#### 5.1.2. Tauopathies

Tauopathies are neurodegenerative disorders characterized by the formation of insoluble brain deposits of tau protein. Studies have shown that the pathophysiology of these diseases is also related to the interaction of tau with RBPs [161]. The role of TIA1 in the development of these diseases has been studied both in vitro and in vivo. For example, the interaction of tau with TIA1 at physiological concentrations is sufficient to trigger LLPS in the presence of RNA, giving rise to highly neurotoxic tau oligomers [100]. In cultured neurons, deletion or depletion of TIA1 reduces the ability of cells to form SGs, inhibits tau misfolding and prevents tau-mediated neurodegeneration [106]. Contrastingly, in brain tissue, tau regulates the distribution of TIA1 and is necessary for its interaction with RNA metabolism proteins, accelerating the formation of SGs [106]. The inter-relationship between both proteins has also been studied in a murine model of tauopathy, wherein it has been observed that TIA1 and other RBPs associated with SGs form and stabilize oligomeric tau accumulations [107]. The existence of a mechanism of TIA1-mediated propagation of toxic tau oligomers and pathological SGs has been proposed [161,162]. In addition, TIA1 depletion also decreases pathological synaptic mRNA alternative splicing events [108]. Although TIA1 depletion increases tau fibrillation, it generally leads to decreased neurodegeneration, increased life expectancy and improved memory in animals [107]. However, depletion or deletion of TIA1 in these models also intensifies neuroinflammatory processes in advanced stages of the disease, suggesting that TIA1 acts as a translational repressor that mitigates inflammation in the central nervous system during chronic stress produced by tauopathy [97]. Taken together, these studies open the door for new pharmacological treatments aimed at decreasing the formation of SGs, which could have beneficial effects on tauopathies (Figure 5 and Figure 6).

#### 5.1.3. Spinal Muscular Atrophy

Spinal muscular atrophy (SMA) is caused by deletions or mutations of the survival motor neuron 1 (SMN1) gene. Its cognate gene, SMN2, cannot compensate for this loss due to the predominant skipping of exon 7 during splicing of its mRNA, which involves TIA1. In a recent study analyzing the effect of TIA1 KO in a murine model of mild SMA [109], the authors verified that although TIA1 is involved in Smn2 splicing, it is not an essential regulator of this event, as was previously believed to be the case in humans [93,163], and its deletion does not significantly affect exon-7 skipping [109]. However, sex-specific effects are evident in this model, as TIA1-/- females show increased body weight, with the caveat that low SMN levels are associated with low body weight. Additionally, the involvement of the autonomic nervous system leads to an increase in tail necrosis in females by negatively affecting its vasculature. In males, the most notable effect was the deficient development of the testes, the transcriptome of which also showed changes after TIA1 deletion. The female reproductive organs are not affected. Gene-expression changes in brain tissue also showed sex-specific differences [109] (Figure 5 and Figure 6).

#### 5.1.4. Stress-Related Psychiatric Disorders

Avoidance behaviors are adaptive responses to environmental threats. Those related to fear are particularly necessary for survival but can be detrimental when they occur excessively, as is the case in post-traumatic stress disorder. Recently, Rayman and colleagues studied the physiological role of TIA1 in mouse brain, concluding that it bidirectionally modulates stress-dependent synaptic plasticity in the hippocampus [98]. They also found that TIA1 deletion potentiates conditioned odor-avoidance behavior in female mice, whereas its overexpression in the ventral hippocampus inhibited both contextual fear memory and avoidance behavior in both sexes [98]. Interestingly, the role of TIA1 in the hippocampus is restricted to the processing of stress-dependent aversive memory and not to other tasks dependent on this region [98]. Overexpression of TIA1 in the ventral hippocampus led to alterations in the transcriptome, both in expression and alternative splicing, with a high prevalence of immune-related genes, especially cytokines, which play an important role in the regulation of synaptic plasticity and hippocampal-dependent learning and memory [98]. Subsequently, to confirm the role of TIA1 in human psychopathology, the authors conducted a population-based study that, although limited, identified a significant interaction between SNPs in TIA1 and stressful life events, which are related to the development of pathological anxiety symptoms [164]. These findings point to TIA1 as a potential therapeutic target for the treatment of stress-related disorders (Figure 5 and Figure 6).

#### 5.1.5. Huntington’s Disease

Huntington’s disease (HD) is an inherited neurodegenerative disorder caused by an abnormal expansion of CAG repeats in the huntingtin gene (HTT), which results in polyglutamine expansions in the protein [165]. Mutant HTT is prone to form aggregates that, in turn, alter proteostasis; they affect protein-protein interactions and the proper functioning of those with which they interact. TIA1 likely plays a role in the development of this pathology, as it is one of the proteins that colocalize with HTT in aggregates, and its fibrillation is induced through the Q/N-rich C-terminal domain [110,166]. TIA1 expression might even increase when mutant forms of HTT are expressed [167], but the decrease in the total amount of soluble protein alters the post-transcriptional regulation of the mRNAs with which it interacts, resulting in an overall decrease in translation [166]. Again, the data point to sequestration of TIA1 in pathological inclusions and its loss of function as one of the processes favoring the development of the disease (Figure 5 and Figure 6).

### 5.2. Welander Distal Myopathy

Welander distal myopathy (WDM) is a dominantly inherited autosomal muscular dystrophy that manifests late in life (40–60 years) [168]. Its first symptoms are weakness in the thumb and/or index fingers, which progress to difficulty in extending the remaining fingers, ultimately involving all the small muscles of the hand. It also eventually affects the distal muscles of the lower extremities, hindering fine motor skills and movement [169,170]. Histologically, the skeletal muscle shows myopathic alterations and rimmed vacuoles, as well as filamentous inclusions [168,170,171]. It is found almost uniquely in Sweden and in some regions of Finland. All individuals with WDM share an uncommon haplotype on chromosome 2p13 [111,112,171], where a heterozygous founder mutation (c.1150G > A; p.E384K) was identified in TIA1 [111,112]. Progression of the disease is faster in the few individuals in whom it is found in homozygosis [171]. These seminal studies identified functional defects of SMN2 exon-7 splicing related to motor neuron disease [111] and disassembly-assembly dynamics of SGs [112] associated with the expression of the TIA1-WDM-mutated version. The effects of the p.E384K mutation of TIA1 were analyzed by Carrascoso and colleagues in a cellular model [93]. While the protein participates in the splicing of *SMN2* exon 7, the authors were only able to observe a mild effect of the mutation on the process, in agreement with previous reports [93,111]. Regarding the dynamics of SGs, the authors observed changes in their assembly and disassembly after oxidative stress, as more stable SGs were generated faster with the mutated TIA1 variant [93,112]. Finally, a negative impact on mitochondrial dynamics and pathophysiology was observed [93]. In this context, the formation of pathological SGs and altered mitochondrial homeostasis in cells expressing TIA1-WDM could be partly responsible for the cellular alterations observed in the muscle tissue of patients and might be a potential therapeutic target [93] (Figure 5 and Figure 6).

### 5.3. Tumorigenesis

Although not considered a cancer-driver protein, TIA1 does have a role in tumorigenesis via its capacity to transcriptionally/post-transcriptionally regulate genes belonging to key pathways for tumor development. In this context, TIA1 exerts a dual function, as it has been shown that depending on the processes regulated in different cancers, it can act both as a tumor suppressor and an oncogene [77,78,79,80,83,87,88,90,113,172,173,174,175]. Contrastingly, TIA1 also controls processes that can prevent the development of tumors: it regulates the alternative splicing of *Fas*, an apoptosis receptor [37,38]; it is a translational repressor of TNFα and regulates the expression of cyclooxygenase 2 (COX2), both related to pro-inflammatory processes [41,132]; it regulates alternative splicing of the fibroblast growth-factor receptor (FGFR2) [13]; it affects the expression and can suppress signaling of HIF1α, involved in angiogenesis [65]; and it is involved in alternative splicing or post-transcriptional regulation of the tumor-suppressor genes neurofibromatosis type 1 (NF1), Wilms’ tumor 1 (WT1) and programmed cell death 4 (PDCD4) [172,173,174,175]. In line with these observations and the role of TIA1 as a potential tumor suppressor, knockdown of TIA proteins in HeLa cells increases cell proliferation, tumor growth and invasion [31,89], while its overexpression leads to cell-cycle arrest, cell death and slow xenotumor development in mice [83]. Furthermore, in other types of cancer, such as colorectal and gastric cancer, TIA1 mRNA has been shown to interact with miRNAs (miR-19a and miR487a, respectively), leading to a decrease in its abundance in the cell and consequent pro-tumor effects [77,78]. By contrast, miR-599 activates TIA1 in thyroid cancer and has a positive effect of inhibiting cell proliferation and metastasis [113]. Along these lines, TIA1 has been proposed as a prognostic marker in lung squamous cell carcinoma, colorectal cancer and urothelial cancer, wherein higher TIA1 expression is associated with better prognosis [114,176]. However, in esophageal squamous cell carcinoma, classical Hodgkin’s lymphoma, renal cancer and hepatocellular carcinoma relapses, high levels of TIA1 are associated with worse prognosis [114,177,178]. This has led to the conclusion that in these latter cancers, TIA1 regulates the expression of genes that favor pro-tumor progression, pointing to TIA1 as a potential oncogene and prognostic marker [114,177,178]. Taken together, a better understanding of the mechanisms through which TIA1 increases or decreases gene expression in cancer may have great utility for the use of this protein both as a prognostic marker and as a potential drug target [23] (Figure 5 and Figure 6).

### 5.4. Diabetes

Obesity is a risk factor for the development of type 2 diabetes, and high-fat diets are known to impair the nuclear localization of the transcription factor pancreatic and duodenal homeobox factor 1 (PDX1), which is essential for glucose-induced insulin synthesis [179]. A recent study showed that saturated fatty acids contribute to the mislocalization of PDX1 in pancreatic beta cells by disrupting nucleocytoplasmic transport and stimulating the formation of SGs that sequester PDX1 and other transport factors [180]. As SG assembly is PI3K/EIF2α-dependent, the use of inhibitors against both or deletion of TIA1 in pancreatic beta cells was found to decrease SG formation and improve cell function. These results raise the possibility of using SGs in pancreatic beta cells as targets for the treatment of type 2 diabetes [180] (Figure 5 and Figure 6).

### 5.5. Lipid Metabolism

TIA1 also plays a role in lipid metabolism. Its depletion in nerve tissue increases the expression of genes that are normally induced by fasting and are involved in lipid storage, transport and membrane trafficking and alters lipid-droplet formation [32]. Moreover, in a murine model of spinocerebellar ataxia type 2, it has been possible to show how pathological aggregation of Ataxin-2 (Atxn2) results in inclusions with sequestered TIA1. Among the proteins whose expression is affected by reduced mRNA expression, those of the cholesterol biosynthetic pathway were found to be prominent, resulting in a drastic decrease in their metabolic precursors [181]. It has also been shown that the aggregation or deficiency of TDP43 triggers an increase or decrease in cholesterol levels, respectively [182,183]. Taken together, the data suggest a prominent role of these SG components in regulation of lipid metabolism by mRNAs under stress conditions (Figure 5 and Figure 6).

## 6. Future Perspectives

Throughout this review, we have detailed the most important discoveries related to TIA1 and its mechanistic implications, as well as the related cellular and pathophysiological processes. Although in recent years, there have been great and relevant advances in this regard, there are still many questions that remain to be answered and that deserve more detailed study. For instance, the differential aspects of the a and b isoforms of TIA1 have been scarcely studied. What we know so far suggests putative differential roles of both isoforms in the regulation of constitutive and alternative splicing, growth suppression, ability to act as proto-oncogenes, regulation of proliferation and cell cycles, etc. Analysis of the interactomes of RNAs and proteins associated with each of the cell-, tissue- and species-dependent TIA1 isoforms would help to address these aspects. The generation of tissue- and isoform-specific animal models with loss- and gain-of-function would also provide very useful information regarding the different activities involving TIA1 isoforms. Moreover, since current technology allows it, obtaining single-cell transcriptomic and proteomic expression patterns could provide novel and much more precise information concerning the role of TIA1 in different cell types.

Another feature that remains poorly studied is the one that encompasses the post-translational modification patterns of TIA1, including phosphorylation, methylation, acetylation, sumoylation, etc., which could form another level of regulation of the protein and its isoforms and, consequently, of the processes that they modulate. Finally, profiles of TIA1 gene mutations should also be obtained in order to discern the role of mutated variants in proteostasis or cancer, as is the case of gain-of-function associated with proteotoxic and tumoral responses. This would also be related to the identification of prognostic and therapeutic targets. The ultimate and most important goal should be the development of drugs that enhance or reduce the functionality of TIA1 or interacting proteins, depending on the disease, by modifying their expression patterns or biological activity.
